# Statistical Modelling and Characterization of Experimental mm-Wave Indoor Channels for Future 5G Wireless Communication Networks

**DOI:** 10.1371/journal.pone.0163034

**Published:** 2016-09-21

**Authors:** A. M. Al-Samman, T. A. Rahman, M. H. Azmi, M. N. Hindia, I. Khan, E. Hanafi

**Affiliations:** 1 Department Wireless Communication Centre, Faculty of Electrical Engineering, Universiti Teknologi Malaysia, 81310, Johor, Malaysia; 2 Department of Electrical Engineering, Islamic University Madinah, Madinah, Saudi Arabia; 3 Department of Electrical Engineering, Faculty of Engineering, University of Malaya, Kuala Lumpur, Malaysia; 4 COMSATS Institute of Information Technology, Abbottabad, Pakistan; West Virginia University, UNITED STATES

## Abstract

This paper presents an experimental characterization of millimeter-wave (mm-wave) channels in the 6.5 GHz, 10.5 GHz, 15 GHz, 19 GHz, 28 GHz and 38 GHz frequency bands in an indoor corridor environment. More than 4,000 power delay profiles were measured across the bands using an omnidirectional transmitter antenna and a highly directional horn receiver antenna for both co- and cross-polarized antenna configurations. This paper develops a new path-loss model to account for the frequency attenuation with distance, which we term the *frequency attenuation (FA)* path-loss model and introduce a frequency-dependent attenuation factor. The large-scale path loss was characterized based on both new and well-known path-loss models. A general and less complex method is also proposed to estimate the cross-polarization discrimination (XPD) factor of close-in reference distance with the XPD (CIX) and ABG with the XPD (ABGX) path-loss models to avoid the computational complexity of minimum mean square error (MMSE) approach. Moreover, small-scale parameters such as root mean square (RMS) delay spread, mean excess (MN-EX) delay, dispersion factors and maximum excess (MAX-EX) delay parameters were used to characterize the multipath channel dispersion. Multiple statistical distributions for RMS delay spread were also investigated. The results show that our proposed models are simpler and more physically-based than other well-known models. The path-loss exponents for all studied models are smaller than that of the free-space model by values in the range of 0.1 to 1.4 for all measured frequencies. The RMS delay spread values varied between 0.2 ns and 13.8 ns, and the dispersion factor values were less than 1 for all measured frequencies. The exponential and Weibull probability distribution models best fit the RMS delay spread empirical distribution for all of the measured frequencies in all scenarios.

## Introduction

With the explosive growth of mobile data traffic and the ever-increasing demand for higher transmission speed, the conflict between increased capacity and spectrum shortage has become an issue of critical importance. An attempt to strike a balance between these two important issues has led to the consideration of mobile broadband technology. Mobile broadband networks can be optimized to increase the chances of fulfilling consumers’ ever-growing demands for higher data rates and to support the predicted exponential increase in mobile traffic volume. Sophisticated signal processing techniques along with new spectrum space for a 5G system are needed to mitigate the physical impairments and fully exploit the system capacity [1,2]. Some models proposed in [3,4] have the potential to be applied in 5G networks, including multichannel signal processing for mitigation of intersymbol and inter-channel interference, constrained coding systems, multiuser coding, multichannel detection, and path diversity.

However, a more formidable radio access technology capable of operating in the newly available spectrum space is urgently required to address the current demands faced by wireless carriers for superior overall system performance, which is projected to continue in the coming decades. Hence, a look beyond the usual 3 GHz spectrum space, also known as the microwave band, is required. The 3–30 GHz spectrum is defined as the super high frequency (SHF) band, while 30–300 GHz is assigned to the extremely high frequency (EHF) or millimeter-wave band. Because radio waves in the SHF and EHF bands share similar propagation characteristics, the 3–300 GHz spectrum, with wavelengths ranging from 1 to 100 mm, can be referred to as the millimeter-wave (mm-wave) band [5,6]. The huge bandwidth available in the mm-wave band has led to the invention of what is known today as millimeter-wave communications. Millimeter-wave communication has been introduced as a key candidate technology for the 5G wireless broadband network; it is capable of providing multi-gigabit communication services, such as device-to-device communication (D2D) [7,8], high definition television (HDTV) and ultra-high definition video (UHDV) [9–11]. As today’s cellular providers attempt to deliver high quality, low latency video and media-rich contents on wireless devices via mobile broadband connections, the issue of bandwidth shortage often restrains them, given that current global broadband communications support only the frequency bands between 700 MHz and 2.6 GHz [12,13].

Despite significant efforts by academic and industrial researchers to create robust wireless technologies, they have always faced an overwhelming escalation in demand for capacity and data rates for the currently deployed technologies, brought about by constant advances in computing and communications technologies, and coupled with the emergence of users’ handheld devices and their needs for internet access. This trend is likely to continue, indicating that wireless networks will face a huge congestion problem by approximately 2020; thus, the need to implement new architectures and technologies to serve the long-term requirements and demands of both the service providers and customers is unavoidable [14]. In the history of cellular technology, the life cycle of every generation of cellular systems has been a decade or less, owing to the rapid evolution of communications and computer technologies. For example, resource management over cognitive radio has been proposed as a traffic-offloading solution to local or remote Clouds by opportunistically exploiting a spectrally limited wireless backbone. The developed controller provides hard reliability guarantees to the Cloud Service Provider and is also capable of self-acquiring context information about the currently available bandwidth-energy resources [15,16]. As the fundamental challenges of wireless communications have been narrowed down to capacity and bandwidth [17,18], recent studies suggest that the mm-wave bands could be used to augment the current depleting bandwidth, to free up the already saturated 700 MHz to 2.6 GHz radio spectrum bands, and to create opportunity for more spectrum access for wireless communications [19]. The introduction of cost-effective CMOS technology that operates efficiently in the mm-wave bands, combined with high-gain and steerable antennas at both mobile and base stations, promises to increase the viability of the mm-wave spectrum in wireless communications [20]. Furthermore, mm-wave carrier frequencies support larger bandwidth allocations, which translate into higher speed transmission. Thus, with mm-waves, service providers have a high degree of freedom to expand channel bandwidths far beyond the present 20 MHz channels used by 4G customers [10]. Increasing the bandwidth of a radio channel results in an increase in data capacity and a decrease in access latency for data traffic; thus, internet access and applications with minimal latency requirements can be sufficiently supported [21]. Due to the much smaller wavelength of the mm-wave, new polarization and spatial processing techniques, such as massive MIMO and adaptive beamforming, can be exploited to compensate for the high propagation loss that characterizes mm-wave communications. With this significant gain in bandwidth and new capabilities made available by exploiting the mm-waves, base station (BS) downlinks and backhaul links between BSs can support much greater capacity than existing 4G networks in areas with higher user densities [21]. In addition to gaining high capacity, operators can further exploit the spatial reuse through methodical reduction in cell coverage areas and by implementing new cooperative architectures such as relays, cooperative MIMO, and coordinated interference mitigation schemes between BSs [22]. As BSs become more densely distributed in urban areas, the cost per BS will drop significantly, resulting in more flexible and cost-effective wireless backhaul deployments. Finally, in contrast to traditional spectrum planning schemes employed by numerous existing cellular operators in which the coverage areas of cell sites vary widely using three octaves of frequency between 700 MHz and 2.6 GHz, the mm-wave spectrum will be allocated in a much closer manner, such that the propagation characteristics of different mm-wave bands will be relatively comparable and almost homogenous [23]. In the future, 28 GHz and 38 GHz bands will be available for spectrum allocations with 400 MHz to 1 GHz of bandwidth [24,25]. These bands of frequencies were originally intended for use only for local multipoint distribution service (LMDS) in the late 1990s [26]; however, due to recent advances, they can now be used for cellular mobile as well as for backhaul communications [27]. The common notion in the wireless engineering community that mm-wave spectrum can easily be devastated by rain and atmospheric conditions no longer makes much sense; when one considers that cell sizes in urban environments are now on the order of 200 m, it becomes obvious that mm-wave cellular systems can withstand issues of signal attenuation [23]. The atmospheric absorption and rain attenuation characteristics of mm-wave propagation are presented in [20] and [23]. Atmospheric absorption has just an infinitesimal adverse effect in terms of path loss for mm-waves for cell sizes as small as 200 m, particularly at 6.5 GHz, 10.5 GHz, 19 GHz, 15 GHz, 28 GHz and 38 GHz. Case studies also document that the attenuations caused by atmospheric absorption on a cell of radius 200 m are as follows: less than 0.002 dB at 6.5 GHz and 10.5 GHz, less than 0.004 dB at 15 GHz and 19 GHz, and less than 0.02 dB at 28 GHz and 38 GHz [20]. Another cellular propagation case study conducted in a tropical rain of 100 mm/h over a cell size of 200 m recorded the following observations: less than 0.2 dB at 6.5 GHz, and 1.8 dB at 10.5 GHz, less than 2 dB at 15 GHz and 19 GHz, and less than 4 dB at 28 GHz and 38 GHz [23].

Apart from the prominent work by authors at New York University (NYU) and the University of Texas at Austin (UTA), there are only a handful of publications on propagation studies of the mm-wave bands performed for downlink mobile access and backhaul communications in compact urban environments. Recently, Samsung has committed time and resources for measuring and studying mm-wave channels likely to be deployed in mobile communications in the near future. The NYU Wireless research center has been one of the most active participants in supporting mm-wave technologies; extensive measurements have been conducted at NYU WIRELESS. UTA and NYU have conducted numerous measurements on channel propagation in the mm-wave bands at different urban microcell (abbreviated UMi in the 3GPP standard) and urban macrocell (UMa) environments. For outdoor environments, many measurement campaigns were conducted by NYU on scenarios that studied different aspects and parameters [23,28–47]; the most-inclusive reference on outdoor propagation channels can be found in [6]. The candidate frequency bands investigated by UTA and NYU are limited to the 28 GHz, 38 GHz, 60 GHz and 73 GHz bands. To characterize the channel propagation characteristics, the channel impulse response as well as the power delay profiles are collected at different spatial transmitter-receiver distances which represent the time-variant channel [47]. For indoor channel and propagation measurements at mm-wave bands, many studies exist on the 60 GHz WiGig frequency bands that have been used in short-range communications such as wireless local area networks (WLAN) [48–51]. Peter et al. [52] conducted measurements on the 28 GHz and 82 GHz mm-wave bands in the laboratory and in an anechoic chamber to characterize the performance of mm-wave channel sounders. A vehicle channel-measurement campaign in the 55–65 GHz frequency band for different antenna placements and occupancy patterns is proposed in [53]. However, few studies have been conducted using mm-wave bands to address 5G wireless networks [25,44,54–61]. In [54], frequency domain measurements were conducted in a laboratory using a vector network analyzer (VNA) with 1 GHz bandwidth and 1 ns time resolution to estimate the channel parameters for multipath components (MPCs). In [55], based on their proposed channel sounder (time domain), the authors reported initial modeling results for shopping-mall-like indoor environments. The large-scale path loss and RMS delay spread from wideband (400 MHz bandwidth) measurements in various indoor environments at 11 GHz and 58 GHz were investigated in [25,62]. In [61], the path loss and RMS delay spread were studied and compared for the lower frequency band of 2.9 GHz and the mm-wave band of 29 GHz in an indoor office environment. In [44,56,57,59], measurements were conducted in an indoor office using sliding-correlator channel sounders with 2.5 ns time resolution and an 800 MHz null-null frequency band at 28 GHz and 73 GHz bands. The path-loss model and time dispersion parameters were characterized in these studies. Maccartney et al. [57] proposed the path-loss models to study the effect of cross-polarization based on cross-polarization discrimination (XPD) factor which is estimated using minimum mean square (MMSE) approach. The complexity of the MMSE approach can be avoided using a new approach that is addressed in this work.

Despite all of the work conducted to date, there are still scenarios and frequency bands in which channel modeling is absent. Extensive characterization and modelling are required in these bands to come up with a generalized model. This study is a part of the series of studies to reach a generalized path-loss model for these bands. This work authenticates some of the existing models and also characterize and propose new scenarios. The contributions of this study are fourfold. First, a new path-loss model is proposed to estimate the frequency attenuation, termed as the *frequency attenuation* (FA) path-loss model. Second, extensive indoor propagation channel characterizations are performed for mm-wave bands of 6–40 GHz. The channel characteristics are investigated based on the proposed and well known path-loss models of single- and multi-frequency schemes for co- and cross-polarization antenna configurations. To reduce the computational complexity of MMSE approach for all cross-polarization path loss models, our third contribution comes in the form of a method to estimate the cross-polarization discrimination (XPD) factor for CIX and ABGX path-loss models. This method is based on averaging the *cross-polarization factor* (XPL) for all measurement points at a particular frequency. Our fourth contribution is the small-scale time-dispersion analysis. These parameters are studied based on two main dispersion parameters: root mean square delay spread (RMS) and mean excess delay (MN-EX). Here, the dispersion of MPCs is analyzed based on a proposed factor termed the *dispersion factor*. A statistical analysis of the RMS delay spread is given using experimental data and different distribution models based on cumulative distribution functions (CDF).

The remainder of the paper is organized as follows. The measurement equipment and environment are described in Sections II and III, respectively. Section IV discusses the large-scale characterizations. The path-loss model results and analysis are presented in Section V. Section VI provides an analysis of the time dispersion parameters. The statistical analysis of path loss and delay spread are investigated in Section VII. Section VIII compares our study with some state-of-the-art indoor channels at mm-wave bands. Finally, conclusions are drawn in Section IX.

## Measurement Equipment and Hardware

**Fig 1:**
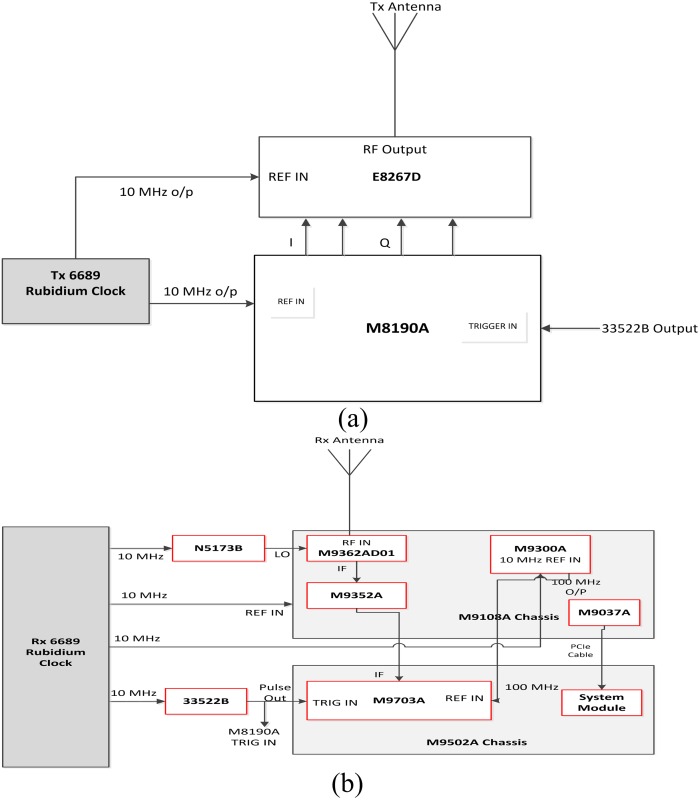
5G Channel Sounder Block Diagram. (a) Tx; (b) Rx.

Using an arbitrary waveform generator (AWG) at the transmitter side to generate a wideband sounding signal and a 12-bit high speed digitizer (bandwidth = 1 GHz) with 1 ns multipath resolution at the receiver side for sounding signal acquisition, extensive mm-wave propagation measurements were conducted at 6.5 GHz, 10.5 GHz, 15 GHz, 19 GHz, 28 GHz and 38 GHz. The Tx and Rx block diagrams are given in Fig 1a and 1b, respectively.

### Transmitter Hardware

The transmitter side of the wideband channel sounder consisted of an arbitrary waveform generator (M8190A), up-converter (E8267D) and rubidium clock (6689). The M8190A was used to generate wideband differential baseband in-phase quadrature (IQ); it could also output direct intermediate frequency (IF) signals with channel sounding. The baseband arbitrary waveform signal provided 1 ns multipath resolution from a pseudorandom binary sequence (PRBS). The E8267D could up-convert this differential baseband IQ into a radio frequency (RF) carrier (up to 40 GHz) with wide modulation bandwidth, and could adjust the output power with its Automatic Line Controller (ALC) circuit. Two 6689 Pendulum clock units (one for Tx, one for Rx) were used in the channel sounder system for synchronization between transmitter and receiver; they could provide a high precision l0 MHz reference signal to all of the instruments with ≤ l*e*-11 accuracy and ≤ 3*e*-11 stability.

The trigger signals could be derived from a rubidium clock or 33522B Function Generation system. The Tx block diagram is shown in Fig 1a. For all measured frequencies (6.5 GHz, 10.5 GHz, 15 GHz, 19 GHz, 28 GHz, 38 GHz), the signal was transmitted with 0 dBm transmitted power through a 3 dBi gain vertically polarized omnidirectional ultra-wideband (0.3–40 GHz) antenna manufactured by Electro Metrics (EM) company. The measurement setup parameters, including frequency settings, are given in Table 1.

**Table 1:** Measurement Setup Parameters.

Carrier frequency (GHz)	6.5	10.5	15	19		28	38
AWCS Signal	10^th^ order PRBS (length = 1024)						
AWCS Chip Rate (Mcps)	1000 Mcps						
AWCS Chip Width (ns)	1						
Digitizer Sampling Rate (Gsps)	3.2						
RF BW (GHz)	1						
Rx LO Power (dBm)	10						
Transmitted Power (dBm)	0						
Gain of Tx antenna (dBi)	3						
Rx antenna Gain (dBi) (Vertical Polarization)	11.5	14.06	14.74		11.6	11.6	15.2
Rx antenna Gain (dBi) (Horizontal Polarization)	11.25	13.86	14.73		11.6	13.1	14.7
Rx Azimuth HPBW (degrees)	38.07	26.96	21.5		38.4	37.6	27.5
Rx Elevation HPBW (degrees)	32.06	23.22	24.1		46.4	44.8	28.3
Height of Tx Antenna (m)	1.7						
Height of Rx Antenna (m)	1.5						
Polarization of Tx	Vertical						
Polarization of Rx	Vertical / Horizontal						

### Receiver Hardware

At the receiver side of the wideband channel sounder, the Rx employed two different types of horn antennas. For the measured frequencies of 19 GHz, 28 GHz and 38 GHz, a wideband horn antenna (18–40 GHz) manufactured by ETS-Lindgren was used. An E-Power-Devices, Inc. wideband horn antenna (2–24.5 GHz) was used for the remaining measured frequencies (15 GHz, 10.5 GHz and 6.5 GHz). The antenna settings at the measured frequencies are given in Table 1. An M9362AD01 down-converter was used to down-convert RF frequencies (up to 40 GHz) to IF, an M9352A hybrid amplifier/attenuator amplified the IF signal, and finally an M9703A 12-bit high speed digitizer of 1 GHz bandwidth (interleaving mode) acquired the IF signal. An N5173B was used for the local oscillator (LO) of the M9362AD01. An M9300A was the Frequency Reference module that took in external 10 MHz and output 10 MHz and 100 MHz standard references; all of the equipment could keep the relative phase stable (phase locked). Because the M9703A accepted only 100 MHz, it was necessary to be use an M9300A. Similar to a Tx 6689, an Rx 6689 Pendulum clock unit also provided a standard 10 MHz reference to all of the instruments. The Rx trigger signal was loaded by a 33522B function generator. Fig 1b shows the Rx architecture block diagram.

### Measurement Environment and Procedure

**Fig 2:**
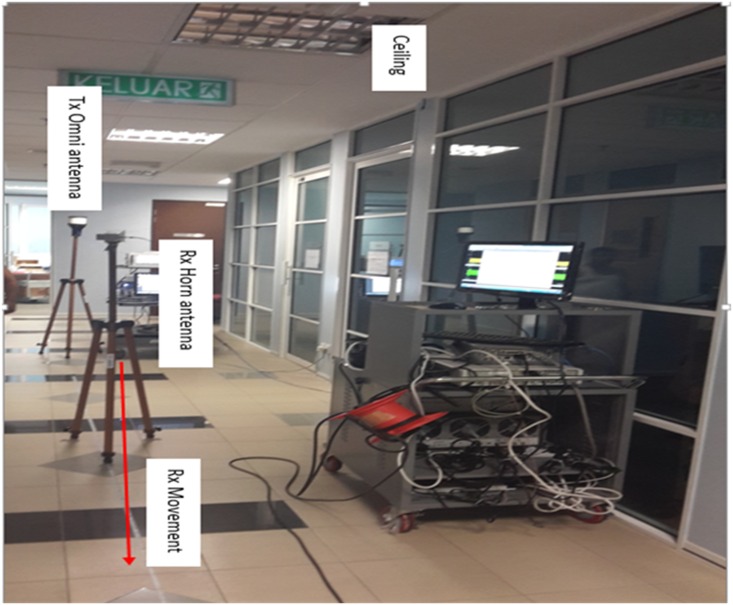
Measurement Setup and Environment.

The ultra-wideband mm-wave measurements were conducted along a corridor on the 15^th^ floor of the Menara Tun Razak Building on the UTM KL campus. This is a 17-story building housing discussion rooms and faculty offices. The size of the corridor testbed is 2.4 m × 40 m, and the ceiling height is 2.8 m. It has plywood and glass doors, and the walls are constructed of concrete, glass and gypsum board. The floor is covered with glazed ceramic tiles, and the corridor ceiling is made of fiberglass materials. Fig 2 shows a pictorial view of the measurement environment. During the measurements, the Tx equipment was stationary and the Rx was moved along the corridor. Tx antennas were placed 1.7 m above the floor to emulate an indoor hotspot on the wall; Rx antennas were placed 1.5 m above the floor (typical handset heights). The measurement was started with the Rx antenna 1 m from transmitter; the received signal was recorded with the Rx stationary at that position. Then, the Rx was moved 1 m farther from the transmitter and the stationary measurements were repeated. The process was repeated at 40 different locations of the Rx, each 1 m away from the previous adjacent location. The measurements were conducted using Line-of-Sight (LOS) scenarios for all frequencies listed in Table 1 with both co- and cross-polarization antenna configurations between the Tx and Rx. For both co- and cross-polarization measurements, the Tx (omnidirectional) antenna was vertically polarized, whereas the RX antenna was vertically polarized for co-polarization (V-V) and horizontally polarized for cross-polarization (V-H). The measurement setup parameters for all measured frequencies are given in Table 1. Based on these measurements, an extensive indoor channel characterization for mm-wave bands was investigated as follows.

### Large-Scale Characterization

The path loss is the main parameter that can be used to describe the large-scale effects of the propagation channel on the received signal. It measures large-scale fading behavior based on power attenuation as a function of distance and frequency. Wireless channel propagation characteristics were investigated based on deterministic, empirical, and stochastic path-loss models [46,63]. However, the most realistic insight into the propagation characteristics of a wireless channel is gained by path loss based on measurements [6,57]. A common path-loss model is defined as [6]:

PlossCI(f,d)[dB]=Ploss(f,d0)+10nlog10(dd0)+Xσ, (1)

where *P*_*loss*_(*f*,*d*) is the path loss at different frequencies with various Tx-Rx separation distance, *P*_*loss*_(*f*,*d*_0_) is the path loss in dB at a close-in (CI) distance, *d*_*0*_, of 1 m, and *X*_*σ*_ is a zero-mean Gaussian-distributed random variable with standard deviation *σ* dB (shadowing effect) [64].

The minimum mean square error (MMSE) is used to calculate the path-loss exponent (*n*) and the standard deviation (S1 Text. Derivative of the studied path loss models). The path loss from co- or cross-polarization or both polarizations (combined polarized) measurements can be estimated from the CI path-loss model. Combined polarization would occur in a practical cellular system with random device orientations [57]. The cross-polarization discrimination (XPD) factor can be added to the CI path-loss model as a special case of cross-polarization propagation. It is defined as the “close-in reference distance with the XPD (CIX) path-loss model” and is given by [57]:

PlossCIX(f,d)[dB]=Ploss(f,d0)+10nlog10(dd0)+XPD+XσCIX, (2)

where *n* is the co-polarization path-loss exponent that is determined from measurements using Eq 1, and *X*_*σ*_^*CIX*^ is the zero-mean Gaussian (in dB) random variable with standard deviation *σ*_*CIX*_ for the CIX model. In the literatures, the XPD parameter is computed using MMSE approach [57].

In this work, the new approach is proposed to estimate the XPD factor which can be used to simplify the CIX model to avoid the computational complexity of the MMSE approach. First, the *cross-polarization factor* (XPL) is calculated as:

$$XPL(f,d)[dB]=P_{loss}{}_{\left(V-V\right)}-P_{loss}{}_{\left(V-H\right)},$$ (3)

where *P*_*loss*(*V*−*V*)_ and *P*_*loss*(*V*−*H*)_ represent the co- and cross-polarization path losses, respectively. Then *XPD* factor can be calculated from Eq 3 by averaging all *XPL* values over distance at carrier frequency *f* that is defined as:

$$XPD(f)=\bar{XPL(f,d)}$$ (4)

The XPD of Eq 4 can be compensated in Eq 2, and the shadow fading (SF) term is calculated by:

XσCIX=PlossCIX(f,d)[dB]−Ploss(f,d0)−10nlog10(dd0)+XPD (5)

In addition to the proposed cross polarization factor method to compute the XPD factor, the work also develops new path loss model named *the frequency attenuation (FA) path-loss model*. The FA path-loss model is given by:

PlossFA(f,d)[dB]=Ploss(fref,d0)+10nreflog10(dd0)+XF(f)+XσFA, (6)

where *P*_*loss*_(*f*_*ref*_,*d*_0_) is the path loss in dB at the close-in distance *d*_*0*_ of 1 m and the reference frequency *f*_*ref*_. The *f*_*ref*_ in this model is defined as the lowest measured frequency using the same calibration environment; *n*_*ref*_ represents the path-loss exponent (PLE) at *f*_*ref*_, which is computed from the CI path-loss model for V-V and V-H antenna configurations. The factor *XF*(*f*) is the frequency attenuation factor in dB, which represents the signal drop due to the frequency, and *X*_*σ*_^*FA*^ is the shadow-fading term with a standard deviation of *σ* dB. The MMSE approach was used to derive the shadowing and frequency attenuation factors. The FA path-loss model is physical-based model and is simple, as is the CI model.

Another famous path-loss model is the *α*, *β* model, which is called floating-intercept (*FI*) model and can be defined as [6]:

$$P_{loss}{}^{FI}\left(d\right)[dB]=\alpha +10.\beta {\text{log}}_{10}\left(d\right)+X_{\sigma }{}^{FI},$$ (7)

where *α* is the floating-intercept in dB and *β* is the slope of the line. Shadow fading is represented by the zero-mean Gaussian random variable *X*_*σ*_^*FI*^ dB with a standard deviation of *σ* dB derived from MMSE closed-form optimization.

The ABG path-loss model is another useful model that can be used to investigate the frequency dependence of path loss in addition to the distance dependence in the CI model. It is given by [29]:

PlossABG(f,d)[dB]=10αlog10(dd0)+β+10γlog10(f/1GHz)+XσABG,  d0= 1 m, (8)

where *α* is the distance-dependence factor of path loss, *β* is an optimized offset, and *X*_*σ*_^*ABG*^ is the shadow fading term. The ABG model is used mainly for co-polarization; it can be used for cross-polarization by using the data set from cross-polarization measurements. The MMSE approach is used to estimate the ABG model. Similar to the CIX model, the ABG model parameters (*α*, *β*, *γ*) can be used for V-H propagation measurements, and the ABGX model is provided as [57]:

PlossABGX(f,d)[dB]=10αlog10(dd0)+β+10γlog10(f/1GHz)+XPD[dB]+XσABGX (9)

Similar to the CIX model simplification method using the proposed XPL Eq (3) and XPD Eq (4), the ABGX models are used with Eqs 3 and 4 for calculating XPD, and then the SF term and its standard deviation are calculated by:

XσABGX=PlossABGX(f,d)[dB]−10αlog10(dd0)−β−10γlog10(f/1GHz)−XPD[dB] (10)

## Path-Loss Model Results and Analysis

### Path Loss for Single Frequency

**Fig 3:**
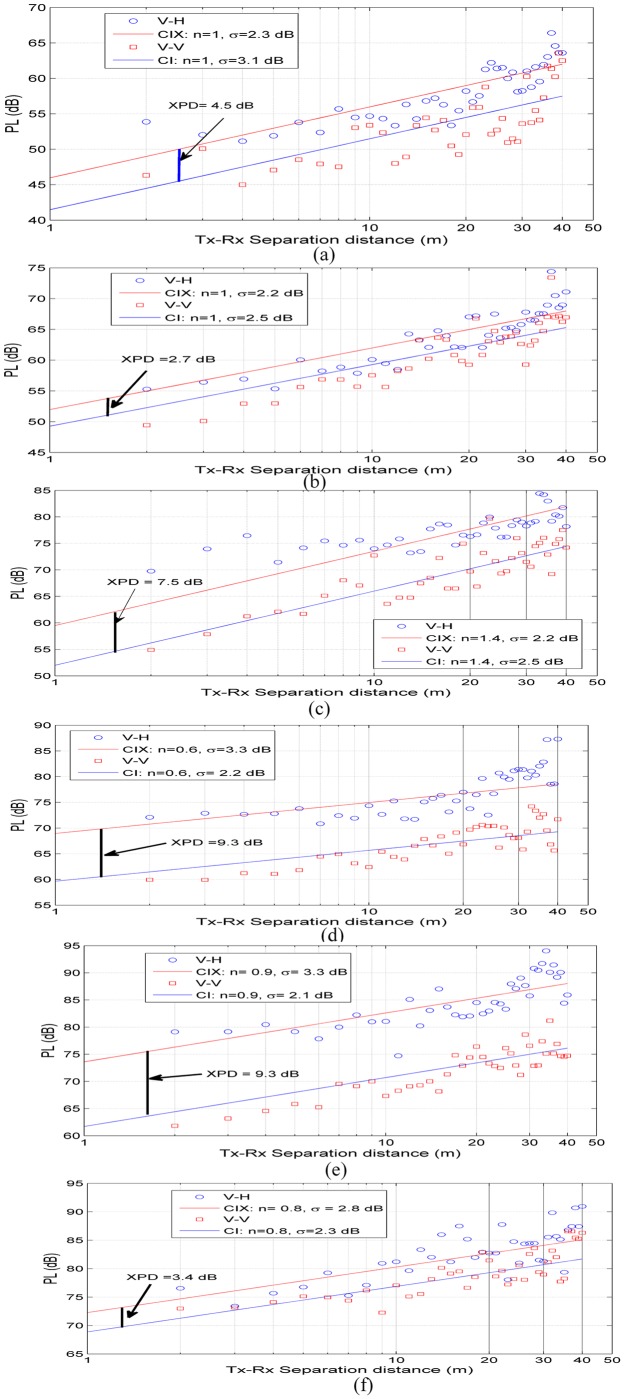
CI (d0 = 1 m) path-loss model for indoor channels in mm-wave bands for V-V and V-H polarization antennas. (a) 6.5 GHz, (b) 10.5 GHz, (c) 15 GHz, (d) 19 GHz, (e) 28 GHz and (f) 38 GHz.

We have investigated different path-loss models based on extensive wideband measurements at various frequencies. The results determine all parameters of the CI, CIX, FA and FI path-loss models for 6.5 GHz, 10.5 GHz, 15 GHz, 19 GHz, 28 GHz and 38 GHz using co-polarization (V-V) and cross polarization (V-H) antenna configurations (Tables A and B in S1 Text). Fig 3a–3f show scatter plots of the path loss and best fit CI model for each frequency (single frequency scheme) at 6.5 GHz, 10.5 GHz, 15 GHz, 19 GHz, 28 GHz and 38 GHz for V-V and V-H antenna polarizations. Table 2 lists the CI parameters for V-V and V-H antenna polarizations at all frequencies. For V-V antenna polarization, the PLEs are 1, 1, 1.4, 0.6, 0.9, and 0.8 at 6.5 GHz, 10.5 GHz, 15 GHz, 19 GHz, 28 GHz and 38 GHz, respectively. The PLEs with V-H antenna polarization are 1.3, 1.2, 1.9, 1.4, 1.8 and 1.1, respectively. These results show that the PLE values for all frequencies, with both V-V and V-H antenna polarizations, are less than the theoretical free space path loss (*n* = 2), indicating that the MPCs from both side walls along the corridor add up constructively, as a waveguide effect; note that the path-loss exponent (PLE) is not frequency dependent. The same phenomenon has been reported at different frequencies in indoor environments [57,60]. The PLE values are identical for 6.5 GHz and 10.5 GHz at both antenna polarizations. Identical PLE values can be shown also at 19 GHz, 28 GHz and 38 GHz for V-V antenna polarization. The standard deviations for V-V and V-H polarizations at 6.5 GHz and 10.5 GHz bands are approximately 3 dB and 2 dB for CI models, respectively, and vary between 2.2–2.8 dB and 2.7–4.4 dB in the rest of the frequency bands for V-V and V-H polarizations, respectively.

**Table 2:** Single frequency CI, CIX and FI path-loss model parameters for all measured frequencies in indoor channels; Freq. and Pol. stand for frequency and polarization, respectively.

Freq. (GHz)	Pol.	CI		CIX			FI			FA		
		PLE	*σ* [dB]	PLE (*n*_v-v_)	XPD [dB]	*σ* [dB]	*α* [dB]	*β*	*σ* [dB]	PLE (*n*_*ref*_)	*XF(f)* [dB]	*σ* [dB]
6.5	V-V	1	3.1	-	-	-	40.7	1	3.1	1	0	3.1
	V-H	1.3	2.3	1	4.3	2.3	44.3	1.1	2.2	1.3	0	2.3
10.5	V-V	1	2.5	-	-	-	45.4	1.3	2.3	1	7.4	2.5
	V-H	1.2	2	1	2.7	2.2	48.5	1.3	2	1.3	8.4	2.8
15	V-V	1.4	2.8	-	-	-	51.9	1.4	2.8	1	15.7	4.1
	V-H	1.9	4.4	1.4	7.5	3.1	63.5	1.1	2.9	1	19.6	6.9
19	V-V	0.6	2.2	-	-	-	56.6	0.9	2.1	1.3	13.4	4.8
	V-H	1.4	3	0.6	9.3	3.3	63.3	1.1	2.8	1	19.4	6.8
28	V-V	0.9	2.1	-	-	-	58.7	1.2	2	1.3	18.3	5.3
	V-H	1.8	3.8	1.8	11.9	3.3	69.1	1.1	3.1	1	26.9	8.4
38	V-V	0.8	2.3	-	-	-	67.9	0.9	2.3	1	25.4	7.9
	V-H	1.1	2.7	0.8	3.4	2.8	70	1	2.7	1.3	25.4	8.5

The CIX path-loss model Eq (2) used the PLE of V-V antenna polarization to estimate the XPD factor. The values of the XPD discrimination factors for all frequencies were estimated for the CIX models as shown in Fig 3a–3f. All parameters of the CIX path-loss model are listed in Table 2. The table shows that the largest XPD factor is 11.9 dB at 28 GHz, implying that the discrimination between cross-polarized signals is strong in this frequency band compared to other bands studied in this environment. The smaller value of the XPD factor is 2.7 dB in the 10.5 GHz band. From the CI path-loss model Eq (1), the PLE (n) for V-H is 1.8 at 28 GHz, which is double that of the V-V CI model PLE (0.8), as shown in Table 2. The PLE (n) for the V-H CI model is 1.2 at 10.5 GHz, which is approximately identical to the V-V CI model PLE of 1.0, as shown in Table 2. From the XPD factor values and PLE values shown in Table 2, it is observed that the XPD factor increases as the discrepancy between the V-V CI model PLE and V-H CI model PLE increases.

**Fig 4:**
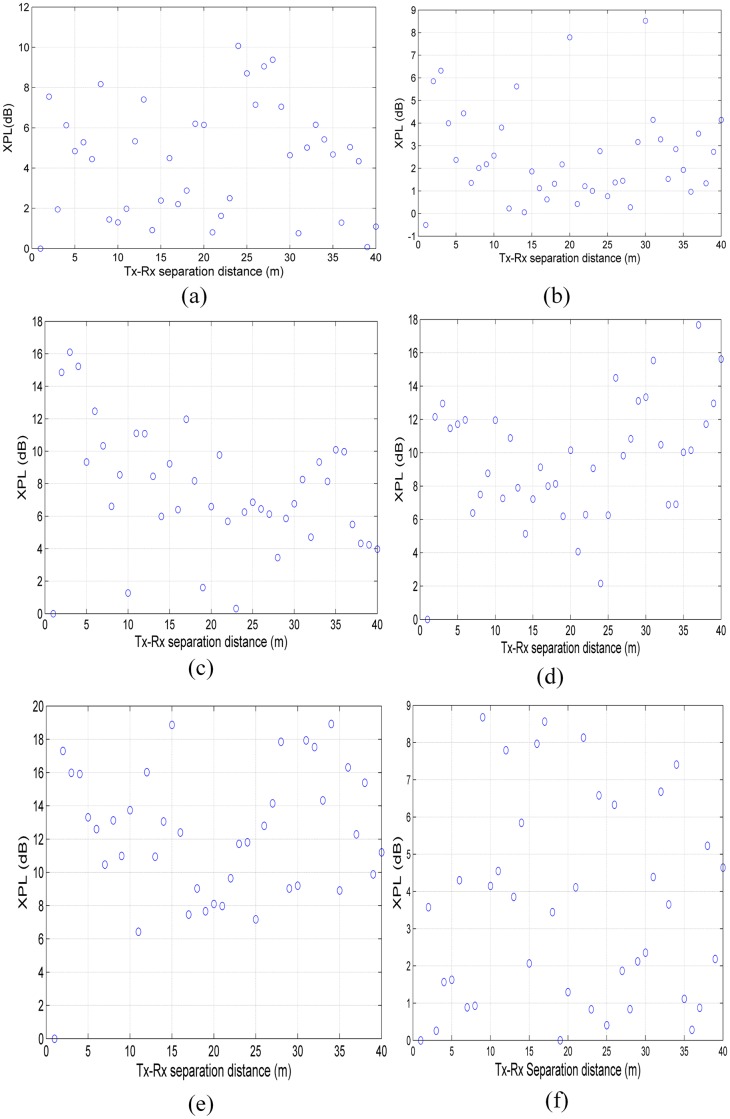
Attenuation Factor (XPL) of cross-polarization antenna configuration versus Tx-Rx separation distance for indoor channels at mm-wave bands. (a) 6.5 GHz, (b) 10.5 GHz, (c) 15 GHz, (d) 19 GHz, (e) 28 GHz and (f) 38 GHz.

The XPL *attenuation factor* models Eq (3) are shown in Fig 4a–4f for all measured frequencies. The XPL factor shows the additional loss of signal due to the cross-polarization antenna configuration at each Tx-Rx separation distance. To reduce the complexity of the MMSE in CIX path-loss model, the XPD discrimination factor is calculated from the proposed methods of Eqs 3 and 4; this indicates that the average value of XPL represents the XPD discrimination factor (Table 2). It is worth mentioning that the estimated XPD values from Eq (4) are identical to the estimated XPD of the CIX models Eq (2). The XPL attenuation has low correlation with Tx-Rx separation distance and measured frequency, as depicted in Fig 4a–4f. That is, no linear relationship exists between *XPL*, *d* and *f*.

The FA path-loss model Eq (6) parameters are listed in Table 2. The reference frequency, *f*_*ref*_, was 6.5 GHz, and PLEs (*n*_*ref*_) are *n*_*V-V*_ (1.0) and *n*_*V-H*_ (1.3) of the CI path-loss model for V-V and V-H polarizations, respectively. The frequency attenuation factor *XF(f)* (dB) values for the 38 GHz band are identical for V-V and V-H frequency attenuation models, respectively. The largest value of *XF(f)* attenuation is 26.9 dB for the 28 GHz V-H model; however, the smallest value is 7.4 dB for the 10.5 GHz frequency band V-V model. The standard deviation of shadow fading in the FA model Eq (6) is greater than that of the CI path-loss model Eq (1) for V-V and V-H antenna polarizations, especially in the higher measured frequencies, as shown in Table 2.

Table 2 lists the FI model parameters; it can be observed that the *α* values deviate from free-space path loss at the reference distance of 1 m for V-V polarizations by 1.4 to 8 dB at different frequencies, implying that the FI model does not physically model the channel. The low values of slope *β* in Eq 7 are approximately identical to the PLE (*n*) values for the V-V CI model in Eq 1 at all measured frequencies. The low values of PLEs in the CI model indicate that the signal gain increases with distance due to constructive interference phenomena of the wireless propagation path; i.e., there is a physical interpretation for the lower values of PLEs in the CI model Eq (1). However, the interpretation of lower values of *β* in Eq 7 is challenging, especially for extrapolation of the FI model outside the measurement range, because there is no physical meaning for the slope *β*. Different thresholding techniques in post-processing methods can also significantly change the parameters in the very sensitive FI model.

The standard deviation values of the FI model are identical to those of the CI model at 38 GHz and 6.5 GHz for both V-V and V-H antenna polarizations, while at the remaining frequencies, there are small deviations (0.1–1.5 dB) between the *σ*^*FI*^ and *σ*^*CI*^ values, as shown in Table 2.

### Path-loss analysis for multi-frequency and combined polarizations

The ABG model represents the frequency path-loss model at various frequencies and includes frequency-dependent and distance-dependent terms. Table 3 provides all of the parameters for the CI, CIX, FA, ABG and ABGX path-loss models for the multi-frequency case. The ABG model shows that the distance dependence factor *α* value is 1.1 for all V-V, V-H and ABGX models. The standard deviation of the ABG model for V-H is more than that of V-V by 4.2 dB. Table 3 shows that the value of the XPD factor in the ABGX multi-frequency model is not high.

**Table 3:** 6.5 GHz, 10.5 GHz, 15 GHz, 19 GHz, 28 GHz and 38 GHz multi-frequency path-loss model parameters for CI, CIX, FA, ABG and ABGX models for indoor channel environments.

**Model**	**Pol.**	**PLE**	**XPD**	***σ***		
**CI**	V-V	0.9	-	3.9 dB		
**CIX**	V-H	0.9	6.5 dB	4.9 dB		
	Pol.	PLE	*XF(f)*	***σ***		
**FA**	V-V	1	13.3 dB	9.2 dB		
	V-H	1.3	16.4 dB	11.3 dB		
	Pol.	*α*	*β*	*γ*	XPD	*σ*
**ABG**	V-V	1.1	15.7	3.1	-	3.2 dB
**ABGX**	V-H	1.1	15.7	3.1	6.6 dB	7.4 dB

The PLEs of the CI multi-frequency model are 0.9 and 1.4 for V-V and V-H, respectively, which are smaller than the FSPL exponent Eq (2) due to the gain from reflected signals. The standard deviation values of the multi-frequency CI models are larger than that of the single frequency by 1–2 dB for both polarizations, shown in Tables 2 and 3. The standard deviation of the CIX multi-frequency is identical to the CI model V-H multi-frequency, indicating that the value of the XPD factor (6.5 dB) is low.

The *XF(f)* values of the proposed FA path-loss model in the multi-frequency scheme are 13.3 dB and 16.4 dB for the V-V and V-H antenna polarizations, respectively. The *XF(f)* multi-frequency attenuation factor values are less than the *XF(f)* factor of a single frequency at higher measured frequencies (38 GHz, 28 GHz, 19 GHz and 15 GHz) for the V-V and V-H polarization measurement. However, at the lower measured frequency of 10.5 GHz, the *XF(f)* multi-frequency values are larger than the single frequency values by 5.9 dB and 8 dB for V-V and V-H, respectively. Hence, it can be concluded that the FA multi-frequency path-loss models are suitable for higher frequency bands. In the multi-frequency scheme, the standard deviation values of the FA model deviate more than 5 dB from those of the CI and ABG models, as shown in Table 3.

Table 4 lists all estimated parameters for the CI, FA and ABG models for combined co- and cross-polarization propagation measurements using all frequencies (multi-frequency scheme). This helps in describing a model in which the receiver orientation is random and the effect of random polarization mismatch is characterized by these parameters. The parameters (*α*, *β*, *γ*) of the ABG model are identical for the multi-frequency V-V measurement and the combined V-V and V-H polarization measurements, as shown in Tables 3 and 4. The standard deviation of the combined ABG model is larger than that of the V-V ABG model by 1.7 dB. The PLE (*n*) of the CI model using combined polarizations is 1.2, which is more than the PLE (0.9) of the V-V CI multi-frequency model by 0.3. Additionally, the standard deviation of the CI combined-polarization multi-frequency model is larger than that of the CI V-V multi-frequency model by 1.6 dB. Furthermore, it is larger than the standard deviation of the ABG combined polarization of multi-frequency by 0.7 dB. The highest standard deviation for combined polarization in a multi-frequency scheme is 10.8 dB for the FA model, where the *XF(f)* factor is 16.6 dB.

**Table 4:** 6.5 GHz, 10.5 GHz, 15 GHz, 19 GHz, 28 GHz and 38 GHz multi-frequency combined polarization path-loss model parameters for CI, FA, and ABG models for indoor channel environments.

**Model**	**PLE**	***σ***		
**CI**	1.2	5.6 dB		
	**PLE**	***XF(f)***	***σ***	
**FA**	1	16.6 dB	10.8 dB	
	***α***	***β***	***γ***	***σ***
**ABG**	1.1	15.7	3.4	4.9 dB

**Fig 5:**
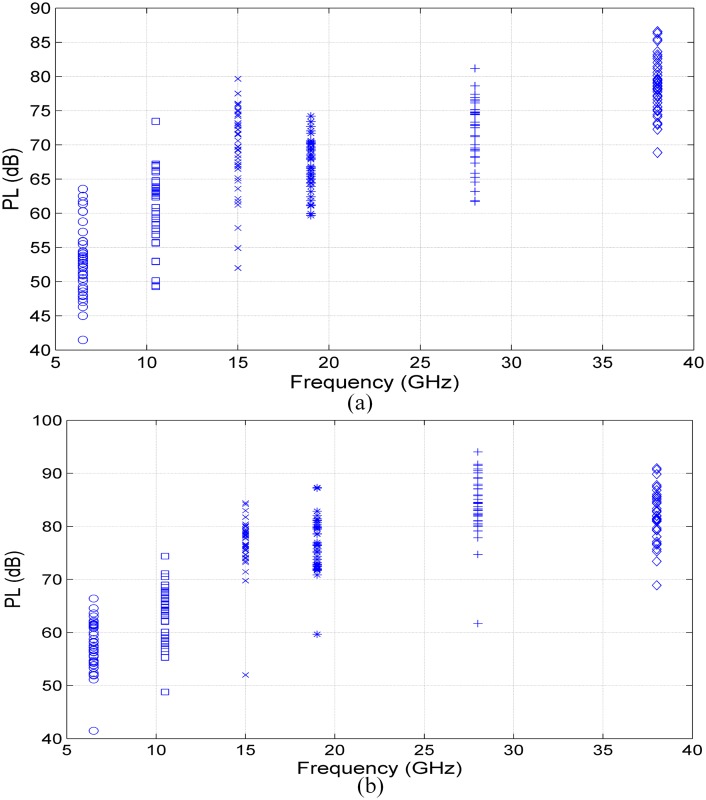
Path loss versus frequency for V-V and V-H polarization measurements for indoor channels of mm-wave bands. (a) V-V and (b) V-H.

Scatter plots of measured PL (dB) versus measured frequencies (GHz) are shown in Fig 5a and 5b for V-V and V-H polarizations, respectively. It can be observed that the PL increases as the frequency increases. However, note that some values of path loss at 15 GHz are larger than those at 19 GHz for V-V polarization measurements. Moreover, some PL values at 28 GHz are smaller than those at 38 GHz. This means that the received signal power depends on the Rx location and the LOS alignment; there are some mismatches of the LOS boresight due to the receiver movement between locations, and the reflected signal may add constructively at some location at a particular frequency while adding destructively at another. Fig 5a and 5b show that on average, the PL for the V-V polarization measurement is smaller than that of V-H.

## Time Dispersion Analysis

In wireless communication systems, the transmitted signal undergoes reflection, refraction, diffraction and scattering. Hence, it can take multiple propagation paths. The signal arriving at the receiver is the superposition of the various multipath components that differ in magnitude and phase from each other by virtue of the different paths. The power delay profile (PDP) of the received signal provides a good indication of the spread of the transmitted power over various paths. The time dispersion characteristics show the distribution of power relative to the first arriving component. These characteristics are usually quantified in terms of the MN-EX delay and RMS delay spread. To obtain these parameters, the PDP is normalized, and all signals below a specific threshold, x dB relative to the maximum, are forced to be zero for the analysis [65, 66]. In this work, the threshold value used was 10 dB, chosen to remove the noise that varies from one measurement setup to another.

These parameters are computed from the power delay profile as [57]:

$$\tau_{rms}=\sqrt{\frac{\sum_{l}p_{l}\cdot {\left(\tau_{l}-\tau_{{}_{b}}-\tau_{m}\right)}^{2}}{\sum_{l}p_{l}}},$$ (11)

where *τ*_*rms*_ is the RMS delay spread, defined by the square root of the second central moment of a power delay profile, *p*_*l*_ is the power for the *l-*th path, *τ*_*l*_ is the arrival time of multipath components, *τ*_*b*_ is the first path arrival time, and *τ*_*m*_ is the MN-EX delay that can be represented by the first moment of the PDP as:

$$\tau_{m}=\frac{\sum_{l}p_{l}\cdot (\tau_{l}-\tau_{b})}{\sum_{l}p_{l}}$$ (12)

Using Eqs 12 and 13, the dispersion of the signal in a wideband system can be defined by:

$$s_{f}=\tau_{m}/\tau_{rms}$$ (13)

The multipath delay profile decays exponentially if *s*_*f*_ = 1. For *s*_*f*_ <1, the concentration of power is high, indicating that most MPCs arrive early; *s*_*f*_ > 1 indicates that the energy arrives at the mid-point of the power delay profile, not the earliest part.

**Fig 6:**
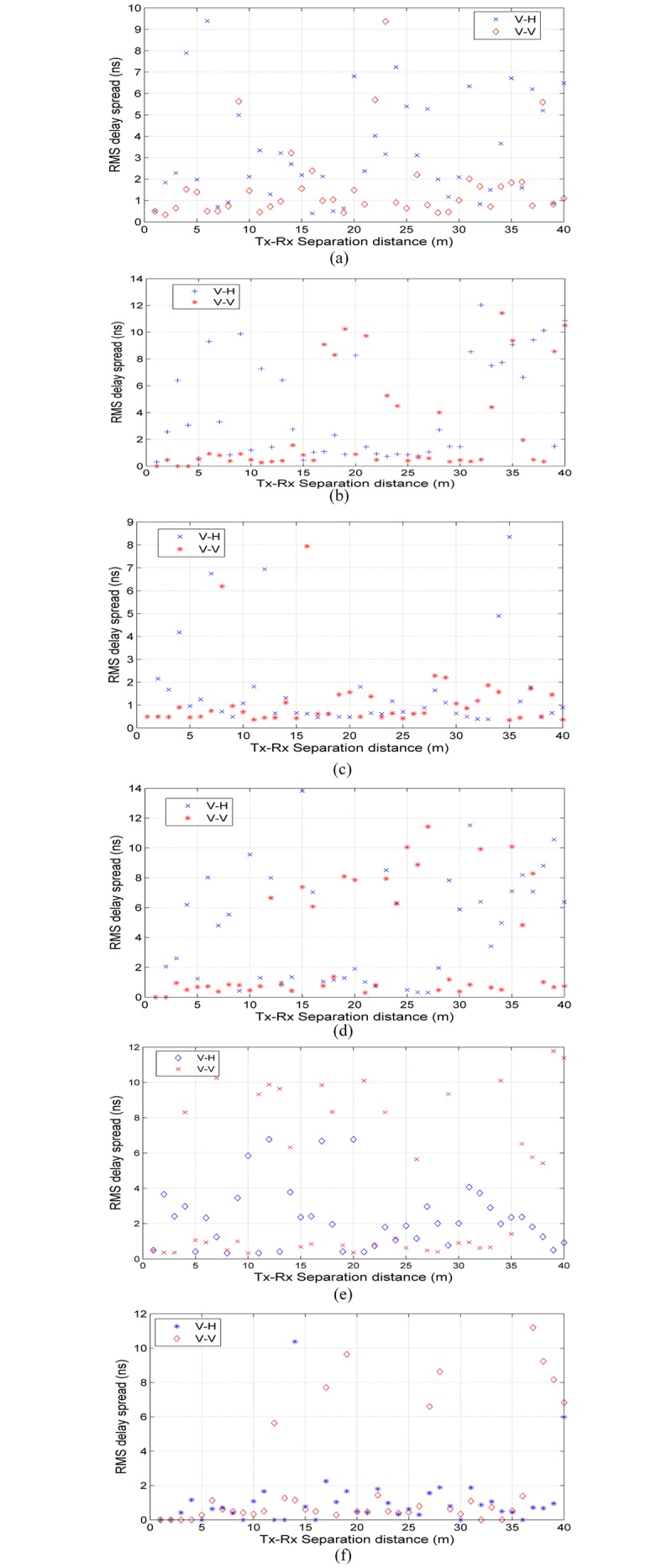
RMS delay spread versus Tx-Rx separation distance for V-V and V-H polarizations for indoor channels for mm-wave bands. (a) 6.5 GHz, (b) 10.5 GHz, (c) 15 GHz, (d) 19 GHz, (e) 28 GHz and (f) 38 GHz.

**Fig 7:**
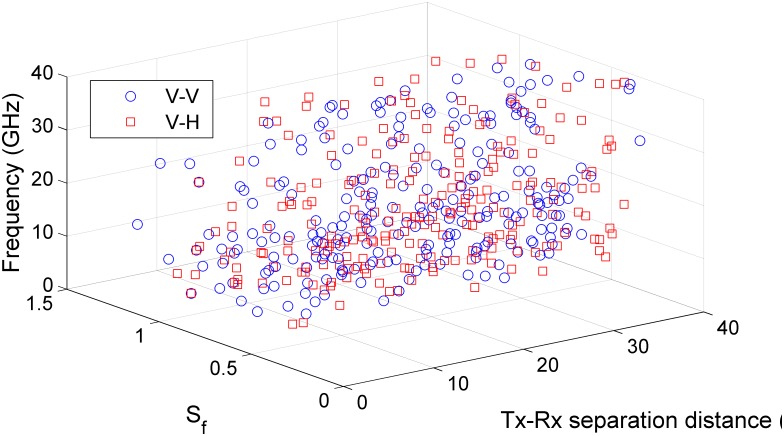
Dispersion factor variation with Tx-Rx separation distance and frequency.

Fig 6a–6f display scatter plots of RMS delay spread with Tx-Rx separation distance at all measured frequencies. From all figures, it can be observed that the relation between RMS delay spread and Tx-Rx separation distance is not consistent. The delay spread depends on the number of arriving multipath components, and the energy and delay of each path at each particular Rx location. The RMS delay spreads of V-V polarization measurements are lower than V-H polarization for over half of the measurements over different locations at 6.5 GHz, 10 GHz and 19 GHz bands as depicted in Fig 6a, 6b and 6d, respectively. The RMS delay spread for V-V is higher than that of V-H at 15 GHz, 28 GHz and 38 GHz as shown in Fig 6c, 6e and 6f, respectively. The maximum excess delays, mean values of RMS delay spreads, MN-EX delays and dispersion factors are listed in Table 5 for all measured frequencies. From Table 5, the maximum excess delay values vary between 24–28 ns for V-V polarization and in the range of 25–40 ns for V-H. The values of maximum excess delay depend on the threshold of post-processing (10 dB for the strongest path in our case study) in addition to the real delay of the measured MPC. The dispersion factors *S*_*f*_ for all Rx measured locations at all measured frequencies are shown in Fig 7. The mean values of *S*_*f*_ are identical for V-V and V-H polarization measurements at all frequencies and are approximately 0.5 to 0.7, meaning that most MPCs arrived early with high power concentration.

**Table 5:** Maximum excess delay, mean values of RMS delay spread, MN-EX delay and dispersion factor for V-V and V-H polarization measurements in indoor channels at mm-wave bands.

Frequency (GHz)	Polarization	MAX-EX (ns)	Mean of MN-EX (ns)	Mean of RMS delay spread (ns)	Mean of *S*_*f*_
6.5	V-V	26	1.2	1.7	0.7
	V-H	31	2.4	3.3	0.7
10.5	V-V	28	1.5	2.8	0.6
	V-H	32	2.8	4.1	0.7
15	V-V	27	0.8	1.2	0.6
	V-H	28	1.1	1.6	0.7
19	V-V	26	3.2	3.3	0.6
	V-H	40	1.8	4.7	0.7
28	V-V	27	2.3	4.3	0.5
	V-H	25	1.7	2.3	0.7
38	V-V	24	1.2	2.3	0.6
	V-H	25	0.7	1.1	0.5

**Fig 8:**
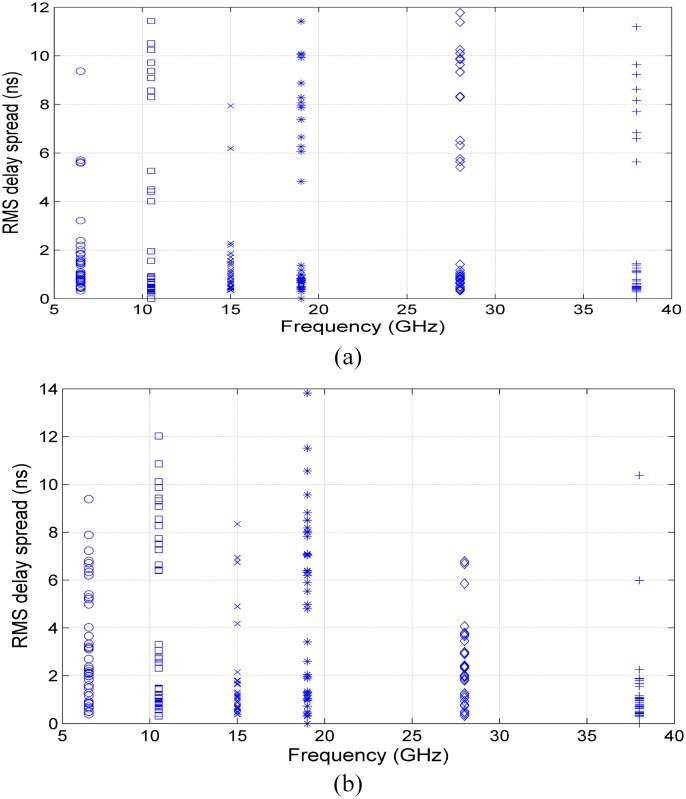
RMS delay spread versus frequency for V-V and V-H polarization measurements for indoor channels of mm-wave bands. (a) V-V and (b) V-H.

The RMS delay spread versus frequency for V-V and V-H are shown in Fig 8a and 8b, respectively. It can be observed that the minimum RMS delay spread values for V-V and V-H at all frequencies were identical and were less than 0.5 ns. The maximum RMS delay spread was 11.7 ns at 28 GHz for V-V polarization and 13.8 ns for V-H polarization at 19 GHz. From Fig 8b, note that most of the RMS delay spread values were less than 2 ns for 15 GHz and 38 GHz and less than 4 ns for 28 GHz. For V-V polarization, most RMS delay spread values at 6.5 GHz and 15 GHz were less than 2 ns.

## Statistical Analysis of RMS Delay Spread

**Fig 9:**
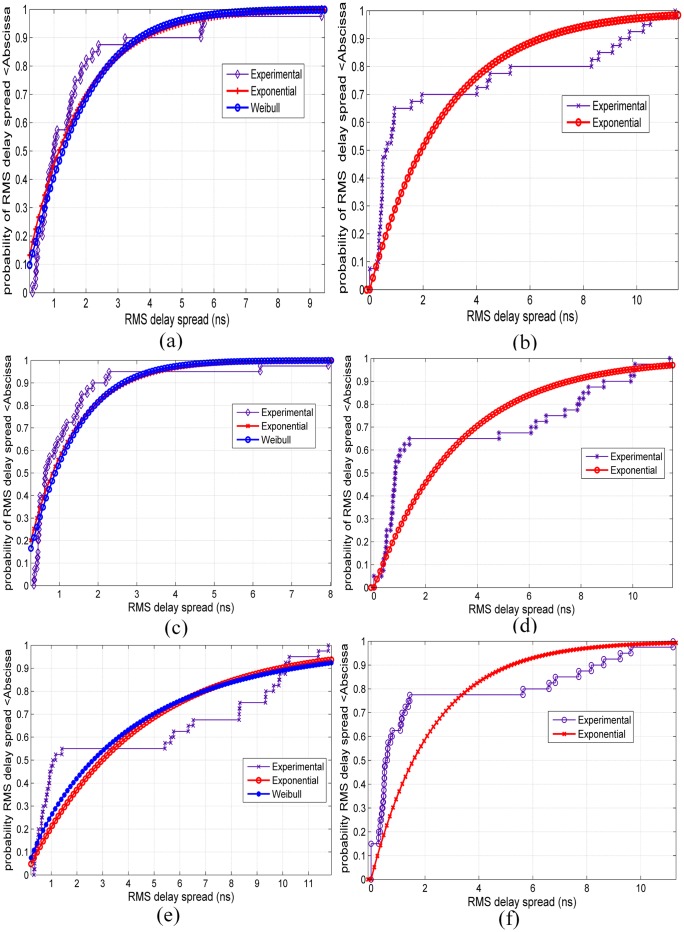
CDFs for RMS delay spreads for V-V polarization at all measured frequencies. (a) 6.5 GHz, (b) 10.5 GHz, (c) 15 GHz, (d) 19 GHz, (e) 28 GHz and (f) 38 GHz.

**Fig 10:**
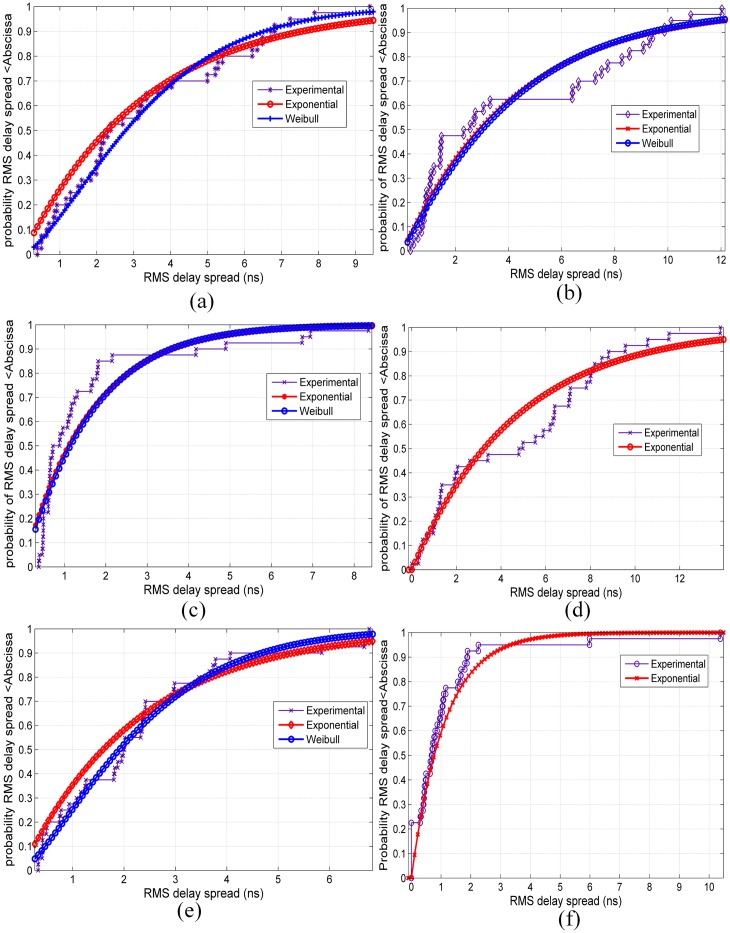
CDFs for RMS delay spreads for V-H polarization at all measured frequencies. (a) 6.5 GHz, (b) 10.5 GHz, (c) 15 GHz, (d) 19 GHz, (e) 28 GHz and (f) 38 GHz.

Statistical analysis is important in studying the distribution of the propagation channel parameters for channel models in wireless communications. Fig 9a–9f show the empirical CDFs of the RMS delay spreads for V-V polarization at all of the measured frequencies along with the Weibull and exponential distribution models of the measured RMS delay spread. The Weibull and exponential distributions best fit the measured RMS delay spread data, as explained in the subsequent paragraphs. Fig 10a–10f show similar CDFs of RMS delay spreads for V-H polarizations at all measured frequencies. For 6.5 GHz, it is apparent that 90% of the energy arrived at the RX between 3 ns and 7 ns for V-V and V-H, as shown in Figs 9a and 10a, respectively. For 10.5 GHz, it is apparent that 90% of the energy arrived at the RX within 10 ns for both V-V and V-H polarizations as shown in Figs 9b and 10b, respectively.

For 15 GHz, it is apparent that 90% of the energy arrived at the RX between 2 ns and 5 ns for V-V and V-H as shown in Figs 9c and 10c, respectively. For 19 GHz, it is apparent that 90% of the energy arrived at the RX within 10 ns for both V-V and V-H polarizations as shown in Figs 9d and 10d, respectively. For 28 GHz, it is apparent that 90% of the energy arrived at the RX within 10 ns and 6 ns for V-V and V-H as shown in Figs 9e and 10e, respectively. For 38 GHz, it is apparent that 90% of the energy arrived at the RX within 8 ns and 2 ns for V-V and V-H as shown in Figs 9f and 10f, respectively.

The best fit to these distributions has been tested using NMSE to estimate the goodness-of-fit (GOF) parameter. The parameters (*μ*, *a*, *b*) of the distributions and the GOFs for V-V and V-H at all frequencies are listed in Table 6; *μ* represents the mean of the exponential distribution, and *a*, *b* are the scale and shape factors of the Weibull distribution, respectively.

**Table 6:** Best fit parameters of the two distributions for the RMS delay spread at 6.5 GHz, 10.5 GHz, 15 GHz, 19 GHz, 28 GHz and 38 GHz, and goodness of fit parameter via NMSE for V-V and V-H polarizations. The NMSE ranges from − ∞ to 1, where − ∞ a poor fit and 1 is indicates a perfect fit.

Frequency (GHz)	Polarization	Exponential Distribution	Weibull Distribution		GOF	
		*μ*	*a*	*b*	Exponential	Weibull
6.5	V-V	1.7	1.7	2.2	0.62	0.63
	V-H	3.3	3.3	5.6	0.37	0.37
10.5	V-V	2.8	-	-	0.27	-
	V-H	4.1	4.1	14.9	0.13	0.13
15	V-V	1.2	1.2	1.2	0.72	0.72
	V-H	1.6	1.6	2.3	0.62	0.63
19	V-V	3.3	-	-	0.22	-
	V-H	4.7	-	-	0.07	-
28	V-V	4.3	4.3	24.7	0.06	0.05
	V-H	2.3	2.3	3.0	0.56	0.56
38	V-V	2.2	-	-	0.37	-
	V-H	1.1	-	-	0.67	-

For 6.5 GHz, the exponential and Weibull distributions provide good fits to the RMS delay spread with identical GOFs of 62% at V-V and 63% at V-H antenna polarizations, as shown by Table 6. From Table 6 and Figs 9c and 10c, it can be observed that the exponential and Weibull models provide the best fits to the RMS delay spread at 15 GHz for V-V and V-H polarizations with 72% GOFs. Additionally, both of the tested distributions can fit the RMS delay spread with 57% GOFs for V-H polarization at 28 GHz; however, the worst GOF (0.05) appears at 28 GHz for V-V polarization.

## Comparison of the Extracted Propagation Parameters with Other Indoor mm-wave Results for 5G Wireless Networks

In this section, the extracted parameters of the path-loss models and RMS delay spreads presented in this paper are compared with the previously reported indoor propagation channel models for LOS scenarios. Due to the inherent differences in the modeling methodologies, e.g., the threshold employed in the post-processing algorithms and the range of measurements, these parameters may not be directly comparable. However, the effects of the environments on the channel characteristics can be observed from the similarities and contrasts in different propagation models. The path-loss exponent, standard deviation, RMS delay spread and some auxiliary parameters in this work are compared with some values from the literature in Table 7.

**Table 7:** Comparison of propagation studies for path-loss models and RMS delay spreads for indoor channels at mm-wave frequency bands.

Source	Distance Range (m)	Frequency Range (GHz)	PLE (*n*)	*α*(FI) (dB)	*β*(FI)	*α*(ABG)	*β*(ABG)	*γ*	*σ*_*CI*_, *σ*_*FI*_,*σ*_*ABG*_(dB)	*τ*_*rms*_ (ns)
Deng et al. [56]	4.1–21.3	28, 73 BW = 0.8	1.1–3.5	_	_	_	_	-	1.7–9	(4.1–21.2) as mean values
Lei et al. [58]	< = 30	28 BW = 1	1.2–2.2	-	-	-	-	-		42.8–57.9
Deng et al. [59]	4.1–21.3	28, 73 BW = 0.8	1.1–4	52.3–100.5	0.2–2.3	0.5–1.9	10.1–32.2	2.4–3.6	0.7–10.6, 0.6–11.7, 1–9.8	-
Haneda et al. [68]	1–10	60, 70 (BW = 4,5)	-	-	-	-	-	-	-	2–20
MacCartney et al. [57]	4.1–21.3	28, 73 BW = 0.8	1.1–3.5	60.4–101.1	0.5–1.6	0.9–1.1	17.7–47.1	2.5–3.5	1.8–8.6, 1.6–15.8, 1.8–14.2	0.5–143.8
Zhou et al. [69]	1–30	15 BW = 1	-	-	-	-	-	-	-	10–259
Eras et al.[60]	1–40	NA	-	-	-	-	-	-	-	6.54–28.84 as mean values
Kim et al.[25]	< 40	11 BW = 0.4	0.36–1.5						0.96–1.9, -, -	< 50
Ours	1–40	6.5, 10.5, 15, 19, 28, 38 BW = 1	0.6–1.9	40.7–70	0.9–1.4	1.1	15.7	3.1–3.6	2–5, 2–3.1, 3.2–5	0.1–13.8

In Table 7, the values of the propagation parameters are reported within a range (lower-upper); this is because the propagation studies of the listed works used different LOS scenarios (single frequency, multiple frequencies, vertical and horizontal and combined antenna polarizations, environment partitioning, directional and omnidirectional models). The lower ranges of PLEs (*n*) are identical for all studies. In this study, the lower range of PLE is 0.6 at 19 GHz frequency for V-V polarization, indicating more gain from MPCs (added up). The largest upper PLE Eq (4) was reported at 73 GHz for V-H polarization in an open plan (large hall) indoor environment [67]. However, in this work, the smallest upper PLE (1.9) was given at 15 GHz (V-H polarization). For our case, this value indicates that the PLEs for all of our LOS cases at all frequencies have FSPL exponents lower than 2 due to constructive interference and the wave-guiding effects of radio wave propagation along the corridor. Furthermore, the lower discrimination effect of cross-polarization measurement reduces the signal drop for V-H polarizations. For the FI model, the value of *α*_*FI*_ depends on the frequency but does not represent the FSPL of 1 m. In this work, it varied between 40.7 dB at 6.5 GHz (lowest measured frequency) for V-V to 70 dB at 38 GHz (highest measured frequency) for V-H. It is consistent with the values of *α*_*FI*_ reported in [57], which are 60.4 dB at 28 GHz for V-V (omnidirectional model) and 101.1 dB at 73 GHz for V-H (directional model). In our study, the value of *α*_*FI*_ was 58.7 dB at 28 GHz for V-V polarization and was 52.3 dB at 28 GHz for V-V (open area indoor office) in [56]. The slope value (it does not represent the actual PLE from physical measurement) of FI, *β*_*FI*_, had the lowest value of 0.2 at 73 GHz for V-V polarization (corridor indoor); however, the lower value in our study was 0.9 in the 38 GHz V-V polarization measurement. Note that the values of the ABG model parameters are consistent with those reported in [57,67]. Additionally, the path-loss exponents at 10.5 GHz for our measurement are consistent with the values reported in [25] for V-V and V-H antenna polarization configurations, respectively. Similarly, the RMS delay spread values are also consistent with the mean values of RMS results reported [56,60].

## Limitations and Future Work

The main objective of the proposed method was to model the channel propagation of the 5G candidate band. However, the study was performed in indoor corridor environments at different polarizations to study the impact of different antenna polarizations. More measurements and scenarios need to be investigated to arrive at a loss factor that expresses different corridor cases. As a future study, the impact of different indoor and outdoor environments will be considered to further generalize the path-loss model. Additionally, time-varying dynamic environments such as streets and parking lots will be investigated. A new factor that expresses the angle of arrival and angle of departure gains will be another point to address.

## Conclusion

This paper presented wideband mm-wave indoor propagation measurements at 6.5 GHz, 10.5 GHz, 15 GHz, 19 GHz, 28 GHz and 38 GHz for co-polarization and cross-polarization antenna configurations. Channel characteristics such as path-loss models for single and multi-frequency, RMS delay spread, MN-Ex and RMS delay spread statistics were presented and modelled. A new path-loss model is proposed to account for frequency attenuation with distance; the model is termed as the *frequency attenuation* (FA) path-loss model. In this model, a frequency-dependent attenuation factor *XF*(*f*) is introduced which directly adds to the CI reference attenuation. Comparison with large-scale path-loss models shows that the close-in free space reference distance models and the FA proposed models are simpler and more accurate and ensure a physical tie to the transmitter power by using the calibration physical distance of 1 m. The CI path-loss models show that the PLE values for this indoor channel vary between 0.6 and 1.0 for V-V polarizations, and between 1.1 and 1.9 for V-H polarizations at all measured frequencies. These are less than the free space path-loss exponent (n = 2), meaning that the multipath components add up constructively due to waveguiding and reflections in indoor corridor environments. The proposed FA models present the frequency attenuation with path loss at a reference distance of 1 m (FSPL of lowest measured frequency, which was 6.5 GHz in these measurements). The largest value of *XF(f)* attenuation was 26.9 dB at 28 GHz for V-H polarization (PLE = 1.3), and the minimum value of *XF(f)* attenuation for measured frequencies above 6.5 GHz was 7.4 dB, found at 10.5 GHz for V-V polarization. The XPL *cross-polarization factor* is proposed to simplify the CIX model to estimate the XPD factor for all measured frequencies. The multipath effects were studied based on time dispersion parameters. An extensive analysis of time dispersion parameters showed that RMS delay spread values were low and that the highest energy arrived with the earliest multipath components. The large-scale path-loss models and time dispersion parameters presented here are important for wideband channel characterization of mm-wave bands at different measured frequencies above 6 GHz. The path-loss models provide valuable information for signal drops in mm-wave bands for candidate frequencies of 5G wireless networks. The time dispersion parameters are very important in designing robust receivers and are used for adaptive transmission techniques.

## Supporting Information

S1 Text Derivative of the studied path loss models. Table A. Path Loss values for Co-polarization (V-V). Table B. Path Loss values for Cross-polarization (V-H).
(DOCX)
