# Supplementary material for: Statistical Modelling and Characterization of Experimental mm-Wave Indoor Channels for Future 5G Wireless Communication Networks
Source: PLoS One. 2016 Sep 21;11(9):e0163034. doi: 10.1371/journal.pone.0163034 (PMC5031408; doi:10.1371/journal.pone.0163034)
Supplement: S1 Text — Table A. Path Loss values for Co-polarization (V-V). Table B. Path Loss values for Cross-polarization (V-H). (DOCX) [file pone.0163034.s001.docx]

**Supporting Information**

**A. M. Al-Samman^1^,*, T. A. Rahman^1^, M. H. Azmi^1^, M. N. Hindia^1^, I. Khan^2,4^, and E. Hanafi^3^**

**^1^Department Wireless Communication Centre, Faculty of Electrical Engineering, Universiti Teknologi Malaysia, 81310 Johor**

**^2^Department of Electrical Engineering, Islamic University Madinah, Saudi Arabia**

**^3^Department of Electrical Engineering, Faculty of Engineering, University of Malaya, Kuala Lumpur, Malaysia**

**^4^COMSATS Institute of Information Technology, Abbottabad, Pakistan**

***Corresponding author**

**E-mail: Ahmedsecure99@gmail.com**

**Contents**

1. **Derivative of the studied path loss models**….…….……………………..…….…3
2. CI path loss model………….………………………………………..…..……3
3. CIX path loss model..………………….……………………………..…….…4
4. FA path loss model…………..………………………………………………..5
5. FI path loss model……………………………………………………………..6
6. ABG path loss model…………………...……………………………………..7
7. ABGX path loss model…………………..……………………………………9

2. **Collected Raw Data**………………………………………………….……...…..10

1. **Derivative of the studied path loss models**


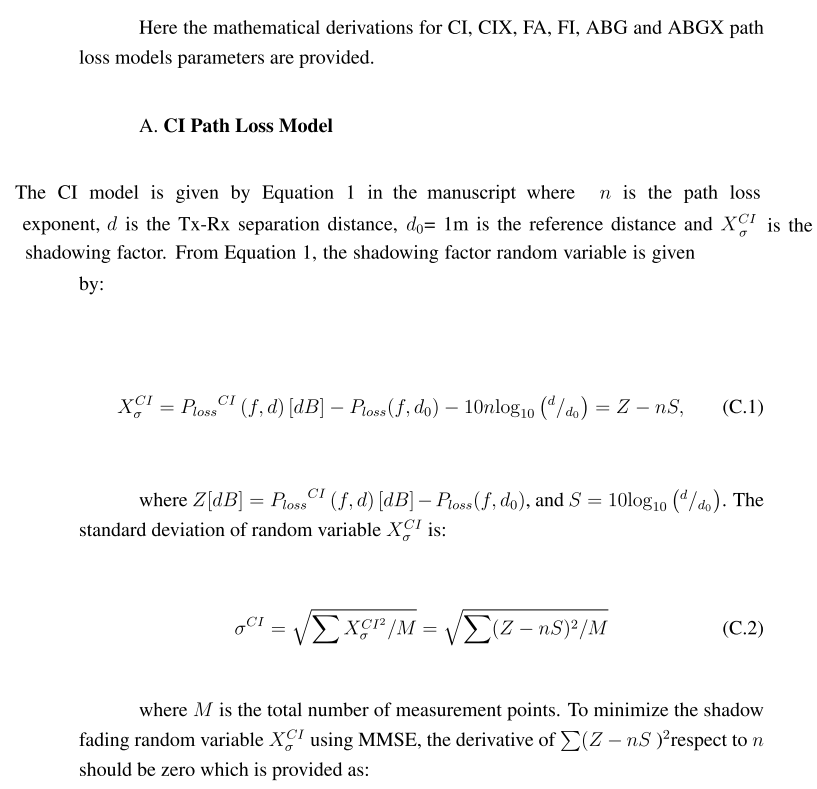


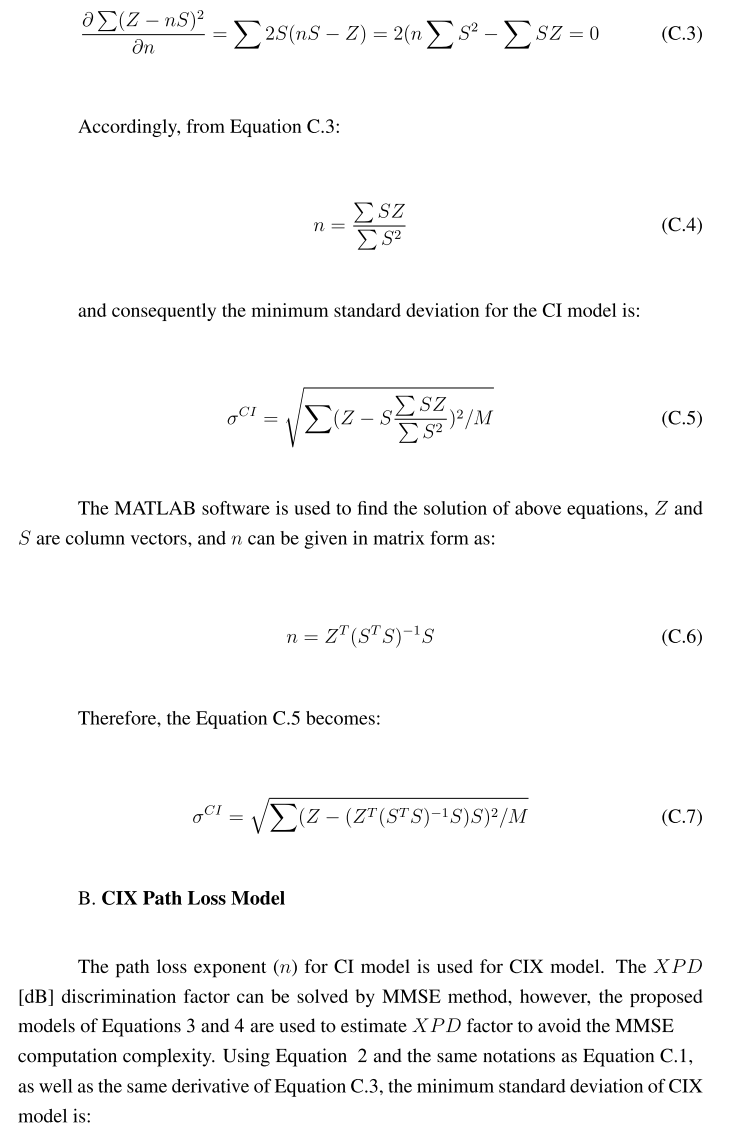


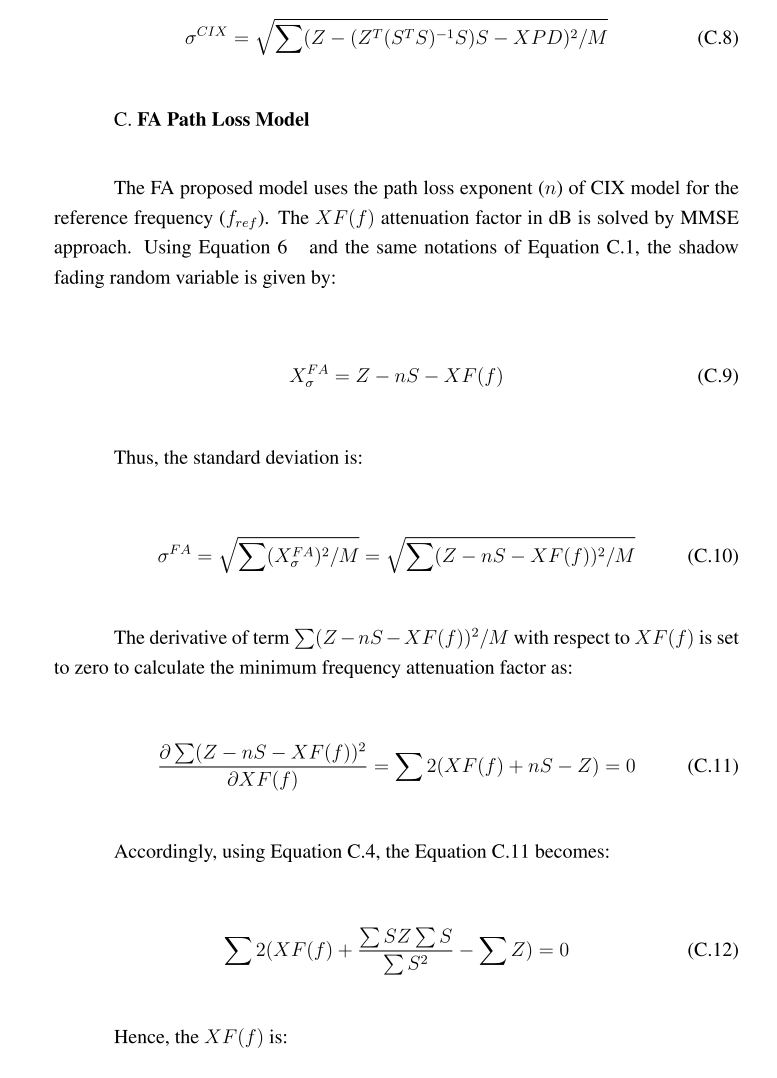


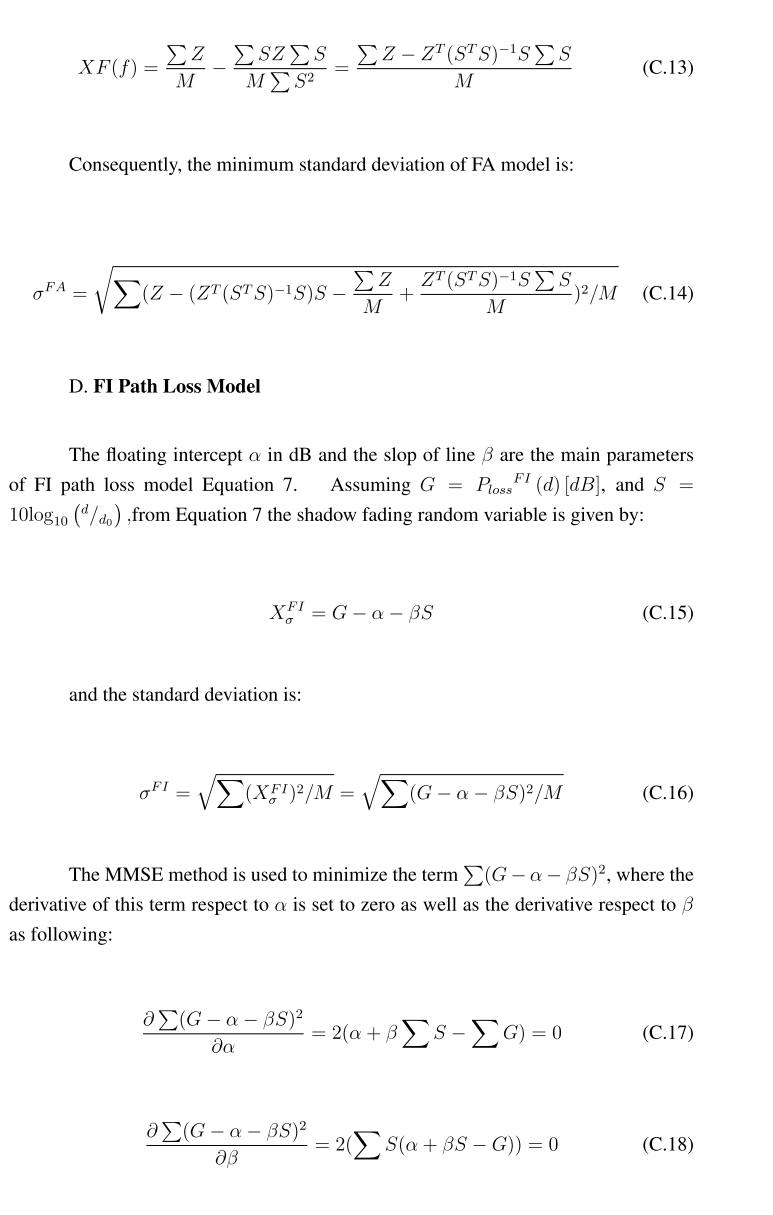

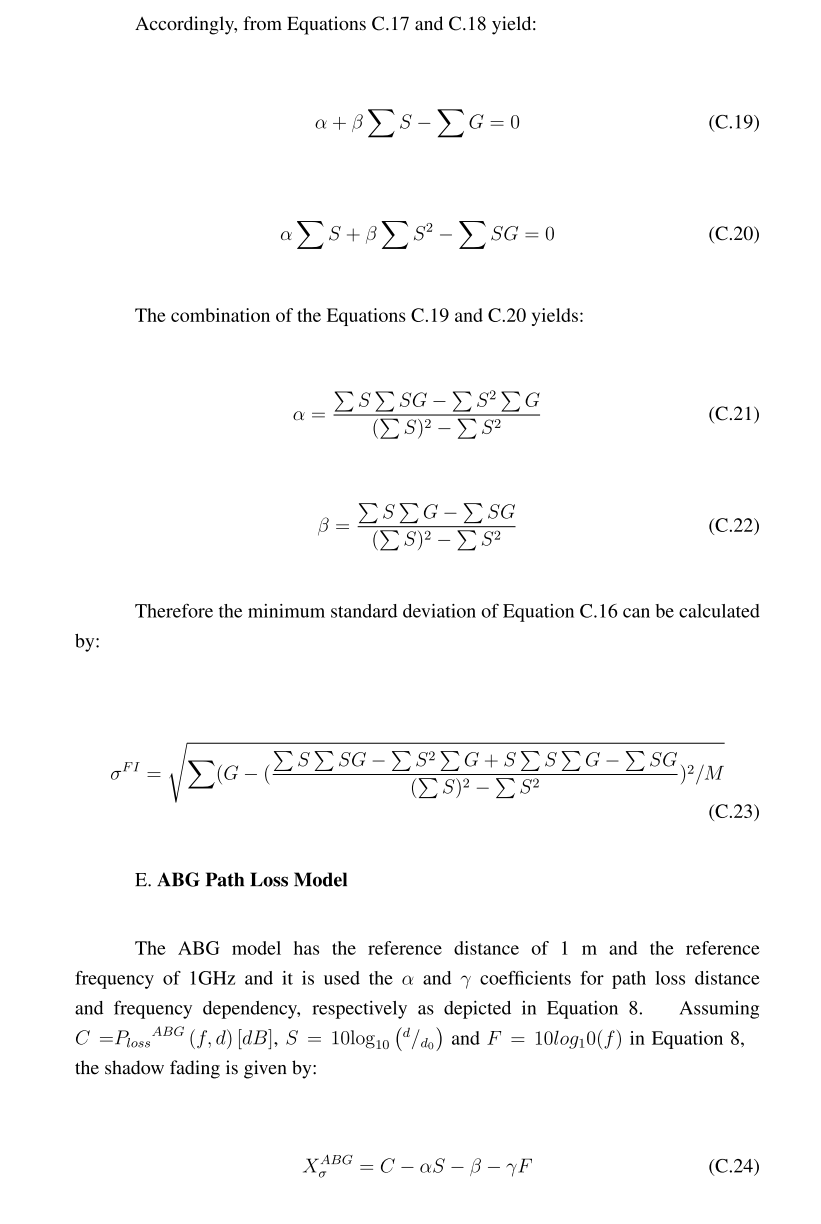

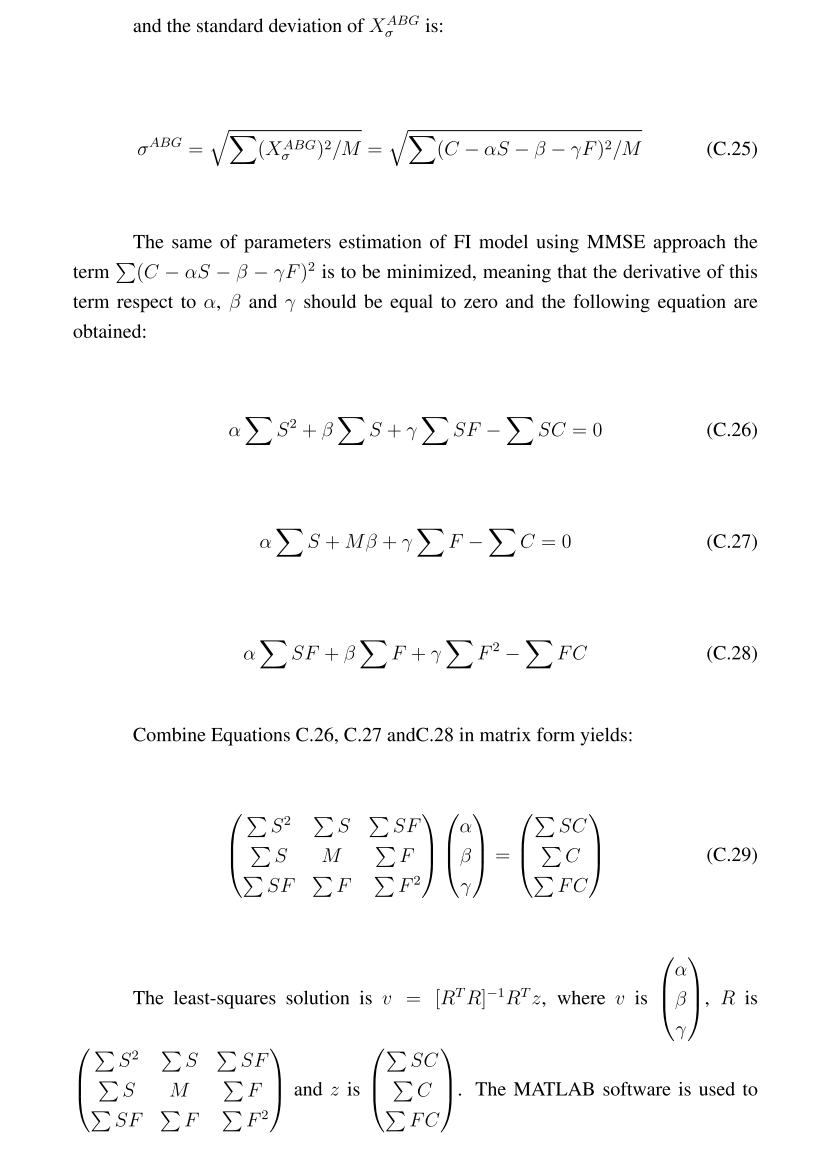

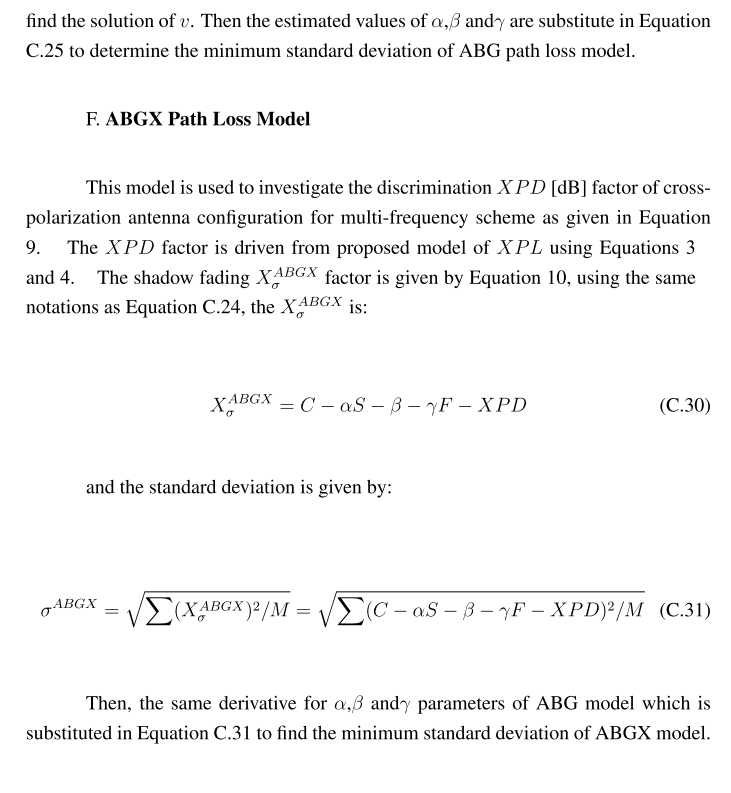


1. **Collected Raw Data**

The Path Loss values for all measured frequencies at all different Tx-Rx Separation at V-V and V-H polarizations are provided in the Tables **A** and **B.** d[m]=[1,2,…,40].

**Table A: Path Loss values for Co-polarization (V-V)**

| **Frequency (GHz)** | **6.5** | **10.5** | **15** | **19** | **28** | **38** |
| --- | --- | --- | --- | --- | --- | --- |
| PL (dB) | 41.47752 | 49.267 | 51.99405 | 59.667 | 61.70216 | 68.8758 |
|  | 46.32115 | 49.42438 | 54.89879 | 59.938 | 61.82362 | 72.981 |
|  | 50.08675 | 50.094 | 57.85433 | 59.9274 | 63.17024 | 73.152 |
|  | 45.00659 | 52.93 | 61.22843 | 61.20987 | 64.56384 | 74.116 |
|  | 47.06031 | 52.96118 | 62.09839 | 61.08094 | 65.84644 | 75.13501 |
|  | 48.52402 | 55.62275 | 61.68483 | 61.81453 | 65.2414 | 74.95591 |
|  | 47.92894 | 56.87237 | 65.12487 | 64.46036 | 69.5293 | 74.38327 |
|  | 47.51695 | 56.84004 | 68.03187 | 64.93122 | 69.12162 | 76.1668 |
|  | 53.0403 | 55.69317 | 67.05562 | 63.14567 | 70.00993 | 72.26237 |
|  | 53.38345 | 57.53479 | 72.71893 | 62.39716 | 67.3227 | 77.06013 |
|  | 52.34652 | 55.63862 | 63.59023 | 65.40124 | 68.28522 | 75.10623 |
|  | 48.00106 | 58.23621 | 64.76013 | 64.40258 | 69.08678 | 75.54136 |
|  | 48.91982 | 58.63821 | 64.76013 | 63.90114 | 69.29108 | 78.14268 |
|  | 53.34873 | 63.22538 | 67.46485 | 66.56032 | 70.01369 | 80.14452 |
|  | 54.42625 | 60.21397 | 68.4968 | 67.85812 | 68.16075 | 79.12621 |
|  | 52.7081 | 63.6508 | 72.24392 | 66.6287 | 71.30935 | 79.51098 |
|  | 54.08027 | 63.33132 | 66.49214 | 68.37574 | 74.80748 | 76.62815 |
|  | 50.47576 | 60.82113 | 66.49214 | 65.02134 | 72.88819 | 78.51262 |
|  | 49.25293 | 59.87064 | 74.94167 | 69.1077 | 74.4023 | 82.90904 |
|  | 52.06326 | 59.24262 | 69.69189 | 66.82901 | 76.40808 | 81.4427 |
|  | 55.87015 | 66.7639 | 66.84895 | 69.69149 | 74.49042 | 78.62967 |
|  | 55.90776 | 60.84501 | 73.1742 | 70.19913 | 73.31897 | 79.63149 |
|  | 58.77735 | 63.0336 | 79.65322 | 70.58886 | 72.82665 | 77.22153 |
|  | 52.1321 | 64.72876 | 71.62734 | 70.35711 | 72.48106 | 78.15896 |
|  | 52.70469 | 62.86531 | 69.31466 | 70.42608 | 76.12231 | 80.5855 |
|  | 54.34833 | 63.80458 | 69.73949 | 66.20425 | 75.15508 | 78.03556 |
|  | 50.94868 | 63.84191 | 72.21668 | 70.10533 | 72.93763 | 82.57743 |
|  | 51.48353 | 64.51356 | 75.98613 | 68.64128 | 71.18836 | 83.60566 |
|  | 51.07224 | 62.61692 | 73.14149 | 68.02204 | 78.61618 | 79.37684 |
|  | 53.60085 | 59.26503 | 71.50313 | 68.08026 | 76.56948 | 78.96287 |
|  | 60.25464 | 62.37753 | 70.59953 | 65.83614 | 72.87066 | 81.13925 |
|  | 53.74984 | 63.17724 | 74.44842 | 69.28222 | 72.94296 | 83.164 |
|  | 55.45375 | 66.03578 | 75.07774 | 74.19424 | 77.38858 | 82.01935 |
|  | 54.1241 | 64.70276 | 76.03642 | 73.3723 | 75.11601 | 77.732 |
|  | 57.25699 | 67.00253 | 72.8848 | 72.03027 | 81.17803 | 78.24829 |
|  | 61.73305 | 73.42994 | 69.20362 | 72.69518 | 75.14585 | 86.53166 |
|  | 61.36209 | 66.94518 | 74.90344 | 69.53143 | 76.89638 | 86.547 |
|  | 60.23105 | 67.17024 | 75.79625 | 66.81848 | 74.68408 | 85.45694 |
|  | 63.54034 | 66.23295 | 77.49731 | 65.63283 | 74.52335 | 85.22563 |
|  | 62.508 | 66.95151 | 74.21313 | 71.69111 | 74.72367 | 86.30675 |

**Table B: Path Loss values for Cross-polarization (V-H)**

| **Frequency (GHz)** | **6.5** | **10.5** | **15** | **19** | **28** | **38** |
| --- | --- | --- | --- | --- | --- | --- |
| PL (dB) | 41.47752 | 48.76463 | 51.99405 | 59.667 | 61.70216 | 68.8758 |
|  | 53.87064 | 55.27486 | 69.75994 | 72.09188 | 79.12318 | 76.557 |
|  | 52.03295 | 56.41095 | 73.95396 | 72.88137 | 79.15207 | 73.40958 |
|  | 51.13634 | 56.92491 | 76.45309 | 72.6786 | 80.47654 | 75.68024 |
|  | 51.89962 | 55.33388 | 71.43716 | 72.79315 | 79.15203 | 76.762 |
|  | 53.8061 | 60.05544 | 74.15327 | 73.7823 | 77.84024 | 79.25587 |
|  | 52.37596 | 58.23054 | 75.45797 | 70.84187 | 79.99438 | 75.26895 |
|  | 55.68672 | 58.84957 | 74.63871 | 72.42336 | 82.24089 | 77.09469 |
|  | 54.48319 | 57.87496 | 75.60026 | 71.91196 | 80.99577 | 80.94 |
|  | 54.68551 | 60.09533 | 73.99298 | 74.35564 | 81.06275 | 81.20713 |
|  | 54.32624 | 59.44498 | 74.69804 | 72.65994 | 74.71403 | 79.65581 |
|  | 53.33264 | 58.46312 | 75.84367 | 75.28867 | 85.10748 | 83.335 |
|  | 56.32774 | 64.259 | 73.21049 | 71.80136 | 80.23105 | 81.999 |
|  | 54.26278 | 63.28381 | 73.44886 | 71.69865 | 83.07572 | 85.99088 |
|  | 56.81233 | 62.08026 | 77.7275 | 75.0781 | 87.02865 | 81.1942 |
|  | 57.20219 | 64.77224 | 78.64756 | 75.76468 | 83.70874 | 87.478 |
|  | 56.29034 | 63.95785 | 78.46286 | 76.37395 | 82.27526 | 85.19115 |
|  | 53.35628 | 62.1405 | 74.67298 | 73.15183 | 81.91456 | 81.95813 |
|  | 55.45128 | 62.04427 | 76.55044 | 75.2969 | 82.05851 | 82.91293 |
|  | 58.21139 | 67.03462 | 76.28717 | 76.98176 | 84.50838 | 82.73819 |
|  | 56.67424 | 67.18803 | 76.624 | 73.75547 | 82.46892 | 82.73867 |
|  | 57.53514 | 62.05439 | 78.86055 | 76.48362 | 82.96731 | 87.76309 |
|  | 61.27724 | 64.03862 | 79.97142 | 79.656 | 84.54032 | 78.05454 |
|  | 62.20132 | 67.49016 | 77.88026 | 72.51136 | 84.2866 | 84.74194 |
|  | 61.40854 | 63.63523 | 76.1729 | 76.67685 | 83.29888 | 80.9923 |
|  | 61.49642 | 65.18161 | 76.18791 | 80.70217 | 87.9538 | 84.36388 |
|  | 60.00115 | 65.29337 | 78.34457 | 79.93487 | 87.08912 | 84.44552 |
|  | 60.86553 | 64.79211 | 79.44057 | 79.48071 | 89.03819 | 84.44446 |
|  | 58.11812 | 65.77928 | 79.00394 | 81.13343 | 87.64902 | 81.49607 |
|  | 58.23958 | 67.79225 | 78.27259 | 81.41273 | 85.76832 | 81.318 |
|  | 61.01704 | 66.52111 | 78.8625 | 81.37524 | 90.80661 | 85.52904 |
|  | 58.76689 | 66.45997 | 79.16382 | 79.76191 | 90.47957 | 89.84503 |
|  | 61.60386 | 67.56518 | 84.41675 | 81.07609 | 91.7104 | 85.67295 |
|  | 59.54702 | 67.55368 | 84.17762 | 80.28042 | 94.03675 | 85.14081 |
|  | 61.93267 | 68.93325 | 82.97863 | 82.05258 | 90.08631 | 79.36319 |
|  | 63.0251 | 74.39402 | 79.18105 | 82.84718 | 91.45659 | 86.814 |
|  | 66.40061 | 70.48341 | 80.398 | 87.20618 | 89.18283 | 87.42152 |
|  | 64.573 | 68.50966 | 80.12391 | 78.53199 | 90.07608 | 90.68708 |
|  | 63.61708 | 68.96545 | 81.73509 | 78.59556 | 84.39768 | 87.41341 |
|  | 63.59815 | 71.09306 | 78.18251 | 87.31217 | 85.9273 | 90.94648 |
